# Trend of estimated glomerular filtration rate during ombistasvir/paritaprevir/ritonavir plus dasabuvir ± ribavirin in HIV/HCV co-infected patients

**DOI:** 10.1371/journal.pone.0192627

**Published:** 2018-02-20

**Authors:** Lucia Taramasso, Antonio Di Biagio, Francesca Bovis, Laura Ambra Nicolini, Andrea Antinori, Laura Milazzo, Salvatore Sollima, Guido Gubertini, Fosca Niero, Annalisa Saracino, Raffaele Bruno, Vanni Borghi, Francesca Montagnani, Annamaria Cattelan, Hamid Hasson, Gloria Taliani, Antonella D’Arminio Monforte, Claudio Mastroianni, Giovanni Di Perri, Sara Bigoni, Massimo Puoti, Angiola Spinetti, Andrea Gori, Nicola Boffa, Bruno Cacopardo, Andrea Giacometti, Giustino Parruti, Vincenzo Vullo, Antonio Chirianni, Elisabetta Teti, Caterina Pasquazzi, Daniela Segala, Massimo Andreoni

**Affiliations:** 1 University of Genova (DISSAL), Infectious Diseases Clinic, Policlinico Hospital San Martino, Genova, Italy; 2 Infectious Diseases Clinic, Policlinico Hospital San Martino, Genova, Italy; 3 Biostatistics Unit, Department of Health Sciences, University of Genoa, Genoa, Italy; 4 Clinical Department, National Institute for Infectious Diseases, INMI L. Spallanzani, Rome, Italy; 5 Department of Biomedical and Clinical Science, University of Milan, Milan, Italy; 6 1st Division of Infectious Diseases, ASST Fatebenefratelli-Sacco, Milan, Italy; 7 Institute of Infectious Disease, University of Bari, Bari, Italy; 8 Division of Infectious and Tropical Diseases, IRCCS Policlinico San Matteo, Pavia, Italy; 9 Infectious Diseases Clinic, University Hospital, Modena, Italy; 10 Department of Internal and Specialty Medicine University Infectious Diseases Unit, AOU Senese, Siena, Italy; 11 Department of Infectious and Tropical Diseases, University Hospital, Padova, Italy; 12 Department of Infectious Diseases, San Raffaele Scientific Institute, Milan, Italy; 13 Department of Clinical Medicine, Policlinico Umberto I, Sapienza University of Rome, Rome, Italy; 14 Clinic of Infectious and Tropical Diseases, Department of Health Sciences, University of Milan, Milan, Italy; 15 Infectious Diseases Unit, Sapienza University of Rome, Latina, Italy, and Department of Public Health and Infectious Diseases, Policlinico Umberto I, Sapienza University of Rome, Rome, Italy; 16 Unit of Infectious Diseases, University of Turin, Department of Medical Sciences, Amedeo di Savoia Hospital, Turin, Italy; 17 Division of Infectious Diseases, AO Papa Giovanni XXIII, Bergamo, Italy; 18 Division of Infectious Diseases, AO Niguarda Ca' Granda Hospital, Milan, Italy; 19 Division of Infectious and Tropical Diseases, University of Brescia and Spedali Civili General Hospital, Brescia, Italy; 20 Division of Infectious Diseases, Department of Internal Medicine, San Gerardo Hospital, University of Milan-Bicocca, Milan, Italy; 21 First Division of Infectious Diseases, S. Giovanni di Dio e Ruggi d'Aragona Hospital, Salerno, Italy; 22 Division of Infectious Diseases, Department of Clinical and Experimental Medicine, ARNAS Garibaldi Hospital, University of Catania, Catania, Italy; 23 Infectious Diseases Unit, Department of Biomedical Sciences and Public Health, Marche Polytechnic University c/o Ospedali Riuniti, Ancona, Italy; 24 Infectious Disease Unit, Pescara General Hospital, Pescara, Italy; 25 Department of Public Health and Infectious Diseases, Policlinico Umberto I, Sapienza University of Rome, Rome, Italy; 26 III U.O.C. P.O. Cotugno, AORN Ospedali dei Colli, Naples, Italy; 27 Clinical Infectious Diseases, Department. of Systems Medicine, Tor Vergata University, Rome, Italy; 28 Clinical Infectious Diseases, Sant'Andrea Hospital—Sapienza University of Rome, Rome, Italy; 29 Unit of Infectious Diseases, University Hospital of Ferrara, Ferrara, Italy; National Taiwan University Hospital, TAIWAN

## Abstract

The renal function is a key-issue in HIV/HCV co-infected patients, nevertheless, it has not established so far whether HCV treatment with new direct acting agents could impact on estimated glomerular filtration rate (eGFR) variations. In the present work, we examined the real-life data on renal function that have been prospectively collected in the SIMIT compassionate-use program of ombitasvir/paritaprevir/ritonavir plus dasabuvir (OBV/PTV/r + DSV) in 144 HIV/HCV genotype 1 co-infected patients. The population was 74% male, 30.5% in CDC stage C, with median age of 52 years (48.0–56.5) and median liver stiffness of 7.8 kPa (6.7–9.2). Median baseline eGFR was 102.0 (90.8–108.1), changing to 99.8 (83.5–104.8) at the end of treatment (EoT), and 100.0 (87.3–105.6) 12 weeks after the EoT (FU12), p<0.0001. No patient had grade 3–4 increase of creatinine. At EoT 60/144 (41.7%) patients had ≥ 5% reduction in their eGFR, confirmed at FU12 in 39/60 (65.0%) cases. Longer duration of HCV infection (cut-off 12.9 years), lower HCV-RNA viral load (cut-off 1,970,160 IU/ml) and lower platelet count (cut-off 167,000 x10^6^/L) were significantly associated with eGFR decline at logistic analysis (_adj_OR 2.9, 95%CI 1.0–8.8, p = 0.05; _adj_OR 3.5, 95%CI 1.2–10.4, p = 0.02; _adj_OR 2.8, 95%CI 1.1–6.8, p = 0.03, respectively). After repeating the analysis throughout a mixed model, a higher eGFR decline was highlighted in patients concomitantly treated with tenofovir (p = 0.0001), ribavirin (p = 0.0001), or integrase inhibitors (p <0.0001), with longer duration of HIV (p = 0.0002) and HCV infection (p = 0.035), lower baseline HCV RNA (p <0.0001), previous HCV treatment (p<0.0001), and older age (p<0.0001). In conclusion, our study confirms a good renal safety profile of OBV/PTV/r + DSV treatment in HIV/HCV patients, and the median decline of 2 ml/min in eGFR, albeit statistically significant, is of doubtful clinical significance. The role of aging, concomitant therapies and duration of HIV/HCV infection needs to be further investigated.

## Introduction

New direct-acting antiviral (DAA) agents have radically changed the therapeutic scenario of chronic HCV infection in both mono-infected and HIV co-infected patients [1, 2]. The 3-DAA regimen of ombitasvir, paritaprevir plus ritonavir, and dasabuvir (OBV/PTV/r + DSV) has showed high efficacy in clinical trials in HIV/HCV co-infected patients [3, 4] and recent data on compassionate-use program for OBV/PTV/r + DSV, coordinated by the Italian Society of Infectious and Tropical Diseases (SIMIT), have confirmed high efficacy in real-life setting in HCV genotype 1 infected patients [5]. Moreover, good tolerability without major adverse events attributable to the study drugs has been reported in the same context [5], but an analysis focalized on the trend of the renal function has not been performed yet. In patients co-infected with HIV/HCV the renal safety is an issue of primary interest, as HCV and HIV both constitute risk factors for renal disease. HIV infection may be linked to HIV-associated nephropathy (HIVAN) or favour renal thrombotic microangiopathy, focal segmental glomerulosclerosis and immunecomplexes deposition in the glomerulus [6]. Moreover, some of the drugs used in combined antiretroviral therapy (cART) have possible renal side effects or long-term toxicity [7]. On the same time, HCV infection is linked to a series of immune-mediated glomerulopathies as well as to possible cryoglobulinemia-linked renal vasculitis [8] and hepatorenal syndromes in more advanced stages of liver disease [9]. Nevertheless, little is known on the trend of estimated glomerular filtration rate (eGFR) in patients who clear HCV infection during and after treatment with new DAAs, especially in real life settings [10, 11]. A worsening of renal function after DAA treatment has been reported in patients with cirrhosis, and in those treated with OBV/PTV/r + DSV [10]. Nevertheless, it has not established so far whether HCV clearance is related or not with an improvement in GFR, or if, on the contrary, the combination of antiretroviral drugs and DAAs can even cause a reduction of GFR, throughout a direct mechanism or indirectly, for drug-drug interactions (DDI).

With the aim of analysing the creatinine and eGFR trends in a specific population of HIV/HCV genotype 1 co-infected individuals treated with the same DAA regimen, we examined the data on renal function of the population treated in the SIMIT compassionate-use program of OBV/PTV/r + DSV [5].

## Materials and methods

The SIMIT compassionate-use program provided access to treatment for patients co-infected with HIV and HCV genotype 1 or 4, with or without compensated cirrhosis.

The program enrolled 213 patients at 26 sites. Patients were treated for 12 or 24 weeks based on the absence or presence of cirrhosis. Ribavirin (RBV) was administered twice daily according to body weight and label recommendations [12]. Patients receiving a ritonavir-boosted ART regimen discontinued the ritonavir component of their ART regimen as recommended by the local OBV/PTV/r + DSV label [13, 14].

In this sub-study, the inclusion criteria were: a) Patient is at least 18 years of age, infected with HIV and with HCV genotype 1 (subgenotype 1a, 1b, other) or 4; b) Patient has compensated cirrhosis (Child Pugh score A) OR liver fibrosis ≤F3 (by Metavir or equivalent assessment including those by an alternative technology such as FibroScan^®^) and a condition which contraindicates an IFN based treatment, or clinically significant extra-hepatic manifestation(s) of HCV infection, or rapidly progressive fibrosis OR Patient has a liver transplant and HCV Genotype 1 infection and is at least 12 months post liver transplantation, has had a recent (within 6 months) assessment of liver fibrosis as ≤F2, does not have a diagnosis of fibrosing cholestatic hepatitis; c) Patients has creatinine levels available at baseline (BL), at the end of treatment (EoT) and 12 weeks after the end of treatment (FU12). Duration of HIV and HCV infection was estimated assuming that infection was contracted at June 15th of the year in which the first positive serologic test was reported. Laboratory data were prospectively collected at BL, EoT, FU12 and 24 weeks after the end of treatment (FU24). GFR was calculated according to the CKD-EPI equation [15], while the stage of kidney disease was classified according to KDIGO (Kidney Disease: Improving Global Outcomes) definition [16]. Creatinine increase was classified according to tables for grading the severity of adult and pediatric adverse events of the division of AIDS (DAIDS) [17]. Fibrosis scores were assigned on the basis of the liver stiffness measured by Fibroscan^®^, F1 (<7.1 kPa), F2 (7.1–9.6 kPa), F3 (> 9.6–12.5 kPa), F4 (>12.5 kPa) [5]. The complete dataset used to perform the statistical analysis is available in S1 Table. Quantitative data were presented as medians (1st and 3rd quartile) and categorical data as absolute numbers and percentages. Some variables (i.e. age, HIV and HCV duration, BL HCV-RNA) were dichotomized according to the best threshold obtained from the receiver operating characteristic (ROC) curve analysis [18]. Dichotomization of explanatory variables has the advantage of providing clinically meaningful odds ratios (ORs) with 95% confidence intervals (95% CIs). A logistic regression model was used to assess the impact of baseline characteristics of patients (age, gender, BMI, CDC stage, grade of liver fibrosis, BL HCV-RNA, years of HIV and HCV infection, BL CD4+ T-lymphocytes and nadir, CKD stage), lifestyle habits (smoke, alcohol use), comorbidities (diabetes, hypertension, symptomatic cryoglobulinemia) concomitant treatments in course of OBV/PTV/r + DSV (tenofovir [TDF] and ribavirin [RBV] use), and previous treatment for HCV, on the reduction of at least 5% in eGFR at both EoT and FU12. The same variables were also studied in a logistic regression model in the post-hoc analyses, to investigate if they could influence a ≥5% improvement of eGFR in a subgroup of patients. Factors significant at univariate analysis were added in a multivariate model after stepwise procedure. A two-way repeated measures mixed model ANOVA analysis with Tuckey post-test was run to establish if there was a relationship between eGFR at follow-up and age, hypertension, stage of fibrosis, TDF, integrase strand transfer inhibitors (INSTI), protease inhibitors (PI) and RBV use, previous HCV treatment, years of HCV and HIV infection, CD4+T-lymphocyte nadir and BL HCV-RNA.

The compassionate use of the combination of OBV/PTV/r + DSV was approved on individual base for each patient (AbbVie^®^ named-Patient Program). The full list of Ethical Committees that approved the program is available in S1 File. All patients in the study provided written informed consent. The study was conducted in accordance with the International Conference on Harmonization guidelines, applicable regulations, and the principles of the Declaration of Helsinki.

## Results

Among patients included in the SIMIT compassionate-use program, 144 HIV/HCV co-infected people satisfied inclusion criteria. The population was 74% male (107/144 patients), with median age of 52 years (48.0–56.5), and median CD4+T-cell count of 658 cells /μl (480–929).The median CD4+T cell nadir was 190 cells /μl (74–314). All but three (2.1%) patients had HIV-RNA load <50 copies/ml. According to Centers for Disease Control and Prevention (CDC) definition, 66 (45.8%) patients were stage A, 44 (30.5%) stage B and 29 (30.5%) stage C. All patients had documented HCV genotype 1 infection (1a in 94, 1b in 44, 1a/b in five and 1 not further characterized in one). Median liver stiffness was 7.8 kPa (6.7–9.2) at baseline. Regarding the route of HIV/HCV transmission, the majority of patients (n = 107, 74.3%) reported previous intravenous drug use, 26 (18.1%) unprotected sexual intercourse (5.6% homo/bi-sexual and 12.5% eterosexual), five (3.4%) transfusions of blood components, while in the remaining seven (4.9%) it was unknown. Three patients had more than one risk factor: two patients with previous intravenous drug use and one with previous transfusions of blood components, that also reported unprotected sexual intercourse. Demographic and clinic characteristics of the study population are summarized in Table 1.

**Table 1:** General characteristics of the study population at baseline (BL). Data are expressed as medians (1st - 3rd quartiles) or absolute frequencies (percentage).

Variable	N		Median (q1-q3)	
**Age**		144	52 (48.0–56.05)	
**Years of HCV**		119	17.9 (10.9–20.9)	
**Years of HIV**		142	23.9 (16.9–27.9)	
**BL CD4**		143	658 (480–929)	
**CD4 nadir**		133	190 (74–314)	
**BL HCV-RNA (IU/ml)**		144	1,137,573 (517,764.5–3,041,840)	
**BL HIV-RNA (copies/ml)**		144	0.0 (0.0–0.0)	
**BL AST (IU/L)**		142		48.5 (34–77.5)
**BL ALT (IU/L)**		143		64 (41–99.5)
**BL PLT (x10**^**6**^**/L)**		143		179,000 (145,000–266,500)
**Variable**		**N**		**n (%)**
**Female**		144		37 (25.69)
**Caucasian**		144		144 (100.00)
**Experienced to HCV treatment**		142		65 (45.77)
**BMI**		134		
<20				16 (11.94)
20–25				75 (55.97)
>25				43 (32.09)
**CDC stage**		139		
A				66 (47.48)
B				44 (31.65)
C				29 (20.86)
**CKD stage**		144		
1				111 (77.08)
2				30 (20.83)
3				3 (2.08)
**HCV Genotype**		144		
1a				94 (65.28)
1b				44 (30.55)
1 a/b				5 (3.47)
1 (unknown)				1 (0.69)
**RISK FACTOR**		144		
IVDU				106 (73.6)
Homosexual/ bisexual				8 (5.56)
Heterosexual				18 (12.5)
Hemotransfusion				5 (3.47)
Other/unknown				7 (4.86)
**HABITS AND COMORBIDITIES**				
Smoking		132		76 (57.58)
Alcool use		140		48 (34.29)
Diabetes		144		14 (9.72)
Hypertension		144		34 (23.61)
Symptomatic Cryoglobulinemia		144		11 (7.64)
**CONCOMITANT THERAPIES**		144		
cART				143 (99.31)
RBV				115 (79.86)
TDF				111 (77.08)
INSTI				110 (76.39)
PI				38 (26.39)

N = number of patients for which data was available; n = absolute number of patients with each variable; % = percentage of patients with the specific variable, calculated as n/N; q1 = first quartile; q3 = third quartile; AST = aspartate aminotransferase; ALT = alanine aminotransferase; PLT = platelets; IVDU = intravenous drug use; BMI = body mass index; CKD chronic kidney disease; cART = combined antiretroviral therapy; RBV = ribavirin; TDF = tenofovir; INSTI = integrase inhibitors; PI = protease inhibitors.

The eGFR was available for all 144 patients at BL, at the EoT and at FU12, according to inclusion criteria, while 73/144 (50.7%) patients also had an available follow-up at FU24. Median BL eGFR was 102.0 (90.8–108.1), changing to 99.8 (83.5–104.8) at the EoT, and 100.0 (87.3–105.6) at FU12 (p<0.0001). No patient had grade 3–4 increase of creatinine. The maximum increase of creatinine levels during treatment was 0.35 mg/dl. At the EoT 60/144 (41.7%) patients had ≥ 5% reduction in their GFR, confirmed at FU12 in 39/60 (65.0%) cases. FU24 was available for 30/60 (50%). Of them, 15/30 (50%) had confirmed eGFR decline ≥ 5% also at FU24.

For 39 patients with confirmed eGFR decline both at the EoT and FU12, the possible association among renal function impairment and other comorbidities (hypertension, diabetes), TDF, PI, INSTI or RBV use, as well as baseline characteristics of the patients (age, sex, duration of HIV and HCV infection, HIV stage, baseline HCV-RNA and platelet count, CD4+ T-cell nadir and BL CD4+T-cell count, liver stiffness and previous HCV treatment) was investigated (Table 2).

**Table 2:** Univariate and multivariate predictors of event (eGFR decline > 5%) in the study population (144 patients).

	*OR (95% CI)* *univariate*	*p-value*	_*adj*_*OR (95%CI) multivariate*	*p-value*
Age >45 years*	3.0 (0.8–10.7)	0.09		
***Gender (Male)***	1.2 (0.5–2.9)	0.66		
***Smoke***	0.7 (0.3–1.6)	0.46		
***Alcohol***	1.2 (0.6–2.7)	0.59		
***Diabetes***	1.6 (0.5–5.0)	0.45		
***Hypertension***	1.4 (0.6–3.2)	0.43		
***Symptomatic cryoglobulinemia***	1.0 (0.2–4.0)	0.99		
***CDC stage B vs*. *A***	1.4 (0.6–3.1)	0.44		
***CDC stage C vs*. *A***	0.7 (0.2–2.0)	0.50		
***Grade of liver fibrosis F2 vs*. *F1***	0.5 (0.1–2.2)	0.33		
***Grade of liver fibrosis F3 vs*. *F1***	0.8 (0.2–4.4)	0.83		
***RBV use***	1.6 (0.6–3.7)	0.32		
***TDF use***	1 (0.4–2.4)	0.98		
***PI use***	0.4 (0.2–1.1)	0.07		
***INSTI use***	2.6 (0.9–7.3)	0.07		
***Previous HCV treatment***	0.6 (0.3–1.2)	0.12		
***BMI (<20) vs*. *(20–25)***	0.7 (0.2–2.4)	0.58		
***BMI 2 (>25) vs*. *(20–25)***	0.5 (0.2–1.2)	0.12		
*Years of HCV > 12*.9*	3.1 (1.1–8.8)	**0.03**	2.9 (1.0–8.8)	**0.05**
*Years of HIV > 16*.9*	2.3 (0.9–5.9)	0.10		
***CKD stage 2 vs*. *1***	0.8 (0.3–2.0)	0.61		
***CKD stage 3 vs*. *1***	1.3 (0.1–14.7)	0.84		
Baseline CD4 < = 350*	0.2 (0.3–1.8)	0.16		
***CD4 Nadir < 100 cells/μl***	1.3 (0.6–2.9)	0.48		
*BL HCV RNA < = 1*,*970*,160*	3.4 (1.3–8.8)	**0.01**	3.5 (1.2–10.4)	**0.02**
*PLT < = 167*,000*	4.1 (1.9–8.8)	**0.0004**	2.8 (1.1–6.8)	**0.03**

TDF: tenofovir; RBV: ribavirin; PI: protease inhibitors; INSTI: integrase strand transfer inhibitors; PLT: platelets; OR: odds ratio; _adj_OR: adjusted odds ratio; CI: confidence interval.

*Dichotomized as per ROC

In a multivariate model including duration of HCV infection (cut-off 12.9 years, dichotomized as per ROC), BL HCV-RNA viral load (cut-off level 1,970,160 IU/ml, dichotomized as per ROC), and BL platelet count (cut-off level 167,000 platelets x10^6^/L, dichotomized as per ROC), longer HCV infection and lower HCV-RNA levels were significantly associated with eGFR decline > 5% (_adj_OR 2.9, 95%CI 1.0–8.8, p = 0.05, and _adj_OR 3.5, 95%CI 1.2–10.4, p = 0.02, respectively), as well as lower platelet count (_adj_OR 2.8, 95%CI 1.1–6.8, p = 0.03).

The results obtained without the dichotomizations as per ROC of continue variables (age, BL HCV-RNA, platelet count, transaminases and years of HCV and HIV infection) were also studied and are presented in S2 Table.

To account for possible differences into restricted subgroups of patients, differences among eGFR values were also estimated at all available time-points, including FU24, for all 144 patients throughout a mixed model (Table 3).

**Table 3:** Number of patients tested throughout mixed models and correlation with eGFR decline at different time-points.

Variable		n	correlation with GFR decline (adjusted p)		
			from BL toEoT	from BL toFU12	from BL to FU24
TDF	Y	111	**0.0001**	0.119	0.078
	N	33	0.408	0.686	0.970
RBV	Y	115	**0.0005**	0.151	0.067
	N	29	0.117	0.536	0.998
PI	Y	38	0.994	0.994	1.000
	N	106	**<0.0001**	**0.024**	**0.016**
INSTI	Y	110	**<0.0001**	**0.031**	**0.046**
	N	34	0.918	0.991	0.999
Age >45 years*	Y	120	**0.0001**	**0.032**	0.1096
	N	24	0.4645	0.9969	0.9854
Hypertension	Y	34	0.9961	0.9126	0.9564
	N	110	**<0.0001**	0.0595	0.0984
Metavir stage of fibrosis	F1	117	**0.0028**	**0.0407**	0.4627
	F2	17	0.6405	1.0000	0.9996
	F3	8	0.2521	0.9996	0.1427
Experienced to HCV treatment	Y	65	**<0.0001**	0.2631	0.0739
	N	77	0.2198	0.343	0.8492
HCV since >12.9 years*	Y	80	**0.0035**	0.094	0.1861
	N	39	0.3615	1.0000	0.9961
HIV since >16.9 years*	Y	106	**0.0002**	**0.0034**	**0.0319**
	N	36	0,3547	1.0000	0.9998
Nadir CD4>100 cells/μL	Y	90	**0.0035**	0.2951	0.5598
	N	43	**0.0082**	0.0813	0.2733
BL HCV-RNA< = 1,970,160* IU/ml	Y	98	**<0.0001**	**0.001**	0.0879
	N	46	0.9723	1.0000	0.9739

GFR: glomerular filtration rate; n: number of patients; Y: yes; N: no; BL: baseline; EoT: end of treatment; FU12: 12 weeks after the end of treatment; FU24: 24 weeks after the end of treatment; TDF: tenofovir; RBV: ribavirin.

*Dichotomized as per ROC. Metavir stage of fibrosis is given according to stiffness evaluation by Fibroscan^®^

**Fig 1:**
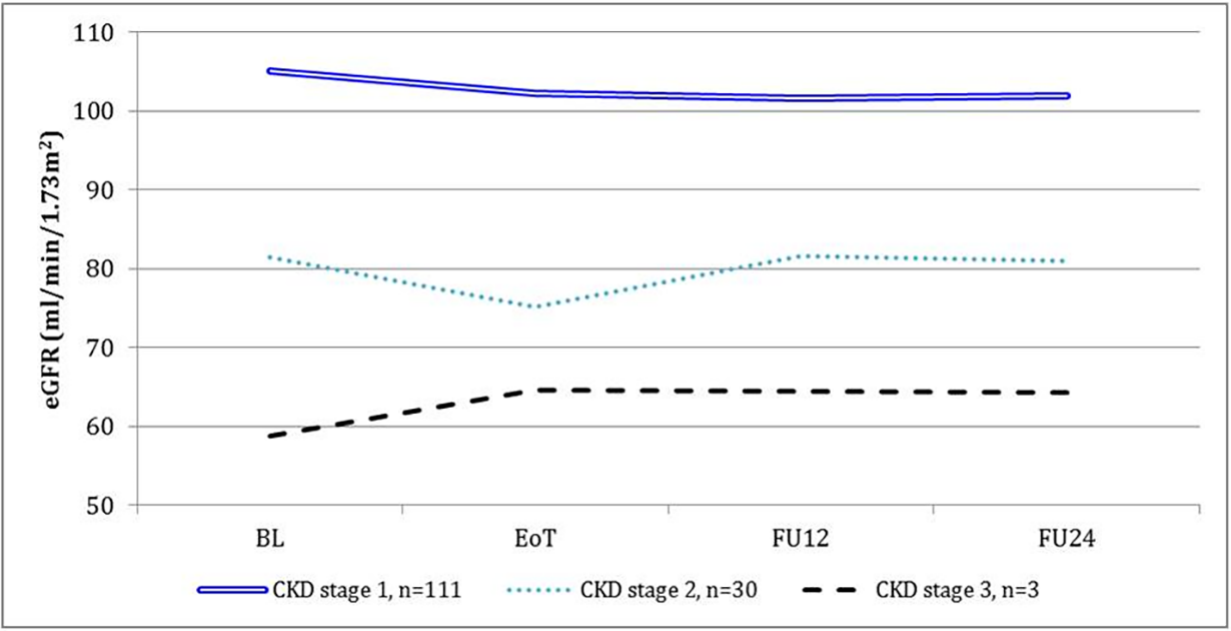
Trend of estimated glomerular filtration rate (eGFR) in the global population, according to GITMO renal stage at baseline (BL). n = number of patients in each chronic kidney disease (CKD) stage. EoT: End of treatment; FU12: follow-up 12 weeks after the EoT; FU24: follow-up 24 weeks after the EoT.

In particular, patients on concomitant treatment with TDF or RBV were at risk for significant eGFR decline compared to people who were not (p = 0.0001 and p = 0.0001, respectively) in the time between BL and EoT, but the difference was not confirmed when comparing BL to FU12 and FU24, nor between values of these time-points compared in pairs. A significant eGFR decline between BL and EoT was also found in patients with at least one of the following characteristics: age > 45 years (p<0.0001), previous HCV treatment (p<0.0001), longer duration of HIV and HCV infections (p = 0.0002 for more than 16.9 years of HIV infection and p = 0.035 for more than 12.9 years of HCV infection), lower baseline HCV-RNA (p <0.0001 for HCVRNA values ≤ 1,970,160 IU/ml), treatment with an INSTI (<0.0001) and not on a PI-containing cART (<0.0001). A longer duration of HIV infection (p = 0.0034), an older age (p = 0.0324), a lower baseline HCV RNA (p = 0.001), INSTI use (p = 0.031) and not being on PI-based cART (p = 0.024) were associated with a significant decrease in eGFR levels in the time between BL and FU12. A longer duration of HIV infection and INSTI use also maintained correlation with a decrease of the eGFR at FU24 (Table 3). The trend of eGFR according to baseline CKD stage is shown in Fig 1. In order to clarify if there was a sub-group of patients who improved their eGFR, a post-hoc analysis was also performed. Overall, 14 patients had confirmed eGFR improvement of ≥ 5% at both EoT and FU12. The logistic analysis revealed that the improvement was more probable in CKD stage 2 and 3 when compared to CKD stage 1 (Odds Ratio, OR, 9.7, 95%CI 2.7–35.2, p = 0.0005 and OR 53.5, 95%CI 4.0–720.1, p = 0.003, respectively), while no significant correlation was found with other factors.

## Discussion

This study analyses the eGFR trend in a large and homogeneous cohort of patients with HIV/HCV genotype 1 co-infection and mild fibrosis prospectively treated with OBV/PTV/r + DSV.

The main findings is that no patient experienced grade 3–4 increase of creatinine levels throughout HCV treatment and FU, and the finding of a median decline of 2 ml/min in eGFR, albeit statistically significant, was of doubtful clinical significance.

Previous trials evidenced the good safety profile and efficacy (higher than 90%) of OBV/PTV/r + DSV among patients with stage IV or V CKD [19]. A very few cases (2/179 patients) of creatinine clearance decrease to <50 ml/min (in a single determination) have been reported in PEARL II study, (3/419 patients, all in treatment with ribavirine) in PEARL III and (3/305 patients, 1 who was in treatment with RBV, and 2 who were not) in PEARL IV [20–22]. Mehta *et al*. [23] have recently analysed the eGFR trend in HCV mono-infected patients treated with OBV/PTV/r + DSV in phase 3 trials SAPPHIRE I/ II, TOPAZ I /II, and RUBY I /II. In this comprehensive analysis, they found a change in eGFR of + 0.39 ml/min in patients enrolled in SAPPHIRE I/II trials (n = 776 patients), with significant improvement in the subgroup of patients with CKD stage 2 and 3. On the other hand, in TOPAZ I/II (n = 2206 patients) and in RUBY I/II trials (n = 78 patients), the overall eGFR decreased, with a significant negative trend (p<0.001) in patients in CKD stage 1 enrolled in TOPAZ I/II trials. Similarly, in our study, we found HIV/HCV co-infected patients had a significant decrease of the eGFR, driven by the high prevalence of CKD stage 1, while, in patients in CKD stages 2 and 3, the eGFR improved. In other trials and registration studies of other DAAs, on-treatment assessments included the evaluations of renal function, but specific analyses on the trend of creatinine clearance have not been performed [22–34]. Some studies had arisen more specifically the issue of renal safety using different criteria for CKD classification and progression as, at present, the evaluation of renal function in course of DAA therapy has not been standardized so far. As a consequence, some studies have reported the numbers of abnormal creatinine rises of any grade [35], while others only those of grade 3–4 [36–38], using different scaling systems. Additionally, the number of patients with an increase in creatinine levels of 0.4 mg/dl or more during treatment has also been used [36,37], or the change in mean creatinine clearance through week 12 [37, 39]. In the general population, the age-related decline in eGFR is generally believed to be less than 1 ml/min each year, i.e. about 1% of eGFR, assuming a filtration rate of 100 ml/min [40], while, in HIV infected patients, a rapid eGFR decline is usually defined by a reduction in eGFR ≥5 ml/min/year in patients with baseline eGFR > 90 ml/min [41]. This corresponds to about 5% decline in patients with normal eGFR (CKD stage 1), while, in patients with baseline eGFR<90 ml/min, a 5% decline permits to take into account also lower absolute changes in eGFR.

We chose to use a decline of eGFR of ≥5% within 12 weeks, i.e. 5-fold higher than what expected in one year period, as a marker of abnormal renal function decline.

Of note, we did not found any creatinine rise of grade >2 or of ≥0.4 ml/min and the reported median decline of 2 ml/min in eGFR, even if statistically significant, has doubtful clinical significance. In fact, current guidelines recommend to refer to a nephrologist in case of eGFR decline by > 25% or to a level < 60 ml/min [42]. Moreover, we cannot exclude that this slight decline could be followed by a late improvement, as previously found in liver transplant recipients with mild chronic kidney disease that achieved SVR after interferon-based regimens [43].

Another remarkable finding of our study is that a ≥5% eGFR decline was related to a longer history of HCV infection and to lower baseline HCV-RNA and platelet count. The correlation with duration of HCV infection could be expected, as, even if the linkage between HCV and CKD is controversial, many evidences underline a possible correlation. In the veteran cohort, HCV infection was found not to have a relation with progressive CKD [44], but, in other large cohorts, both HCV viremic and HCV aviremic individuals were found at increased risk for moderate and advanced CKD compared with HIV-infected subjects who were HCV seronegative [45] and HCV co-infection was found as an independent risk factor for CKD in HIV [46]. On the contrary, the higher probability of eGFR decline in patients with lower BL HCV-RNA was unexpected, and is in contrast with the evidence of higher Odds Ratios for CKD that have been found in patients with higher HCV-RNA [46, 47]. A previous paper on 1,676 HIV/HCV co-infected patients treated for HCV, revealed an incidence of CKD of 5.32 per 1000 person/years of follow-up, without significant differences between patients who achieved sustained virologic response (SVR) and those who not, suggesting a possible minor role for the current viremia on the development of renal injury [48]. On the other hand, higher risks of CKD have been found in patients with detectable HCV RNA than in those who cleared the infection [49] and a higher HCV-RNA has been demonstrated an independent predictor of CKD [50]. We think that these evidences, all together, underline a major role of the global burden of the HCV disease, that is correlated to high HCV-RNA and to years of HCV infection. In the same direction also goes the association between eGFR decrease and lower platelet count, that might suggest a higher probability of renal injury in more advanced stages of HCV disease. Indeed, HCV-RNA is fluctuating and the punctual value at the time of study entry might not represent the real burden of HCV disease in the study population. Another unexpected finding of our study was that the classical risk factors for kidney disease, such as increasing age, male gender, history of smoking, diabetes, and hypertension, had no correlation with impairment of renal function, although it is possible that the effect of such factors could not be appreciable in a relatively young population with low prevalence of diabetes and hypertension.

We also tested eGFR trend within the groups for each measure period, through a mixed model for multiple comparisons that permitted to consider also the interaction between the variables and the time. The results confirmed a role of the longer duration of HCV infection and lower BL HCV-RNA, but also revealed other potential risk factors linked to significant eGFR decline, i.e. TDF, INSTI or RBV use, age > 45 years, previous HCV treatment (mainly IFN-based), and longer duration of HIV infection. Indeed, TDF and IFN are drugs with recognized potential nephrotoxicity, which has also been hypothesized for RBV [51–53] while the effect of INSTI on possible creatinine increase, driven by both dolutegravir and, to a lesser extent, raltegravir [54–55], might have had a role in patients who switched to INSTI to avoid drug interactions during DAA treatment. Moreover, HIV infection and ageing have potential influence on renal function [56]. However, although these results may be of great interest, they must be interpreted with caution, because a significant trend toward reduction was seen in the general population in correlation with time (and in particular between BL and EoT), and it is possible that it is confirmed in any group big enough to allow a reliable analysis. For the same reason, the lack of significance in eGFR decline in patients treated with PI and in those with a history of hypertension do not allow to interpret these factors as protective, but should be rather interpreted as a non-significance, probably due to small number of patients.

This study has several limitations. The lack of a control group constitutes a major limit of this study, as we could not compare the eGFR trend of the study population with that of HIV/HCV co-infected people not undergoing HCV treatment, nor with that of patients treated with other DAA regimens, including those that are now the preferred first-line agents for HCV treatment [57–58]. Moreover, we had no available urinalysis for the study population, and it limits the accuracy of a precise diagnosis of renal injury. Also, we did not have complete data about potentially nephrotoxic concomitant therapies, other than the cART, that could also have played a role, and FU24 was not available for a large part of the study population, so that the possibility of a recovery 24 weeks after the EoT was not adequately investigated. Lastly, we dichotomized the continues variables included in the regression model, as binary covariates offer a simple risk classification into high versus low and, in the regression setting, the interpretation of the impact of a binary covariate on outcome is easier than that for a change of 1 unit in a continuous covariate [59]. However, this approach could have influenced the significance of some results, as it can sometimes lead to biased estimates in regression settings. When categorizing continuous covariates, there is always the possibility of loss of information, possible loss of power to detect actual significance, and possibility of false positive results as well, and using two groups conceals any non-linearity in the relation between the variable and outcome [60].

Despite these limitations, we found that the eGFR decline was significant during HCV treatment, but the same was not found prolonging observation to FU12 in the majority of groups examined through the mixed models. This could lead us to speculate that a temporary nephrotoxicity is possible in course of therapy, but, at least in part, reversible. Low baseline HCV-RNA seems not to be protective in our series, while duration of HCV infection and low platelet count are risk factors for >5% eGFR decline, independently from chronological age. On the contrary, patients with poor renal function at the beginning of treatment had higher probability of renal improvement, even if numbers are too low to draw conclusions.

In summary, our study confirms a good renal safety profile of OBV/PTV/r + DSV treatment in a large homogeneous cohort of HIV/HCV patients. The role of aging, concomitant therapy with TDF, INSTI or RBV, previous HCV treatment and duration of HIV infection need to be further investigated, and studies on larger populations and with longer follow-up are desirable to better clarify the dynamics of renal function in the contest of DAA treatment.

## Supporting information

S1 Table Complete dataset used to perform the statistical analysis. (XLSX)

S2 Table Analysis performed on continue variables without dichotomization. (DOCX)

S1 File Ethical Committees that approved the compassionate use of ombitasvir/paritaprevir/ritonavir + dasabuvir, on individual base for each patient (AbbVie^®^ named-Patient Program). (DOCX)
