# Supplementary material for: Trend of estimated glomerular filtration rate during ombistasvir/paritaprevir/ritonavir plus dasabuvir ± ribavirin in HIV/HCV co-infected patients
Source: PLoS One. 2018 Feb 20;13(2):e0192627. doi: 10.1371/journal.pone.0192627 (PMC5819795; doi:10.1371/journal.pone.0192627)
Supplement: S2 Table — (DOCX) [file pone.0192627.s002.docx]

**S2 Table**: Univariate analysis of predictors of estimated glomerular filtration rate (eGFR) decline ≥ 5% in the study population (analysis performed without dichotomization of continues variables).

|  | OR (95% CI)  univariate | p-value |
| --- | --- | --- |
| ***Baseline HCV-RNA(log_10_)*** | 0.8 (0.6-1.0) | **0.04** |
| ***Years HCV*** | 1.0 (1.0-1.1) | 0.23 |
| ***Years HIV*** | 1.0 (1.0-1.1) | 0.10 |
| ***Age*** | 1.0 (1.0-1.1) | 0.66 |
| ***AST*** | 1. (0.9-1.0) | 0.28 |
| ***ALT*** | 1. (0.9-1.0) | 0.11 |
| ***PLT*** | 1. (1.0-1.0) | **0.03** |

AST: aspartate aminotransferase; ALT: alanine aminotransferase; PLT: platelets.
