# Supplementary material for: Trend of estimated glomerular filtration rate during ombistasvir/paritaprevir/ritonavir plus dasabuvir ± ribavirin in HIV/HCV co-infected patients
Source: PLoS One. 2018 Feb 20;13(2):e0192627. doi: 10.1371/journal.pone.0192627 (PMC5819795; doi:10.1371/journal.pone.0192627)
Supplement: S1 File — (DOCX) [file pone.0192627.s003.docx]

**S1 File: Full name of the IRBs at each of the 26 sites:**

The compassionate use of the combination of ombitasvir/paritaprevir/ritonavir + dasabuvir was approved on individual base for each patient (AbbVie named-Patient Program, approval number not applicable). The complete list of IRBs that have approved the program is:

1. Comitato Etico Regione Liguria, presso Ospedale Policlinico San Martino, Genova, Italy

2. Comitato Etico dell’Istituto Nazionale per le Malattie Infettive Lazzaro Spallanzani, Roma, Italy

3. Comitato Etico Milano Area 1, Sacco Hospital, Milano, Italy

4. Comitato Etico Interregionale, Policlinico di Bari, Bari, Italy

5. Comitato Etico IRCCS Policlinico San Matteo, Pavia, Italy

6. Comitato Etico Ospedale San Raffaele, Milano, Italy

7. Comitato Etico Provinciale di Modena, Modena, Italy

8. Comitato Etico Regione Toscana Area Vasta Sud Est, Ospedale di Siena, Siena, Italy

9. Comitato Etico per la Pratica Clinica dell’ULSS 16 di Padova, Padova, Italy

10. Clinical Infectious Diseases, Department. of Systems Medicine, Tor Vergata University, Rome, Italy

11. Comitato Etico Indipendente, Tor Vergata, Roma, Italy

12. Comitato Etico dell’Università Sapienza di Roma e del Policlinico Sant’Andrea di Roma, Roma

13. Comitato Etico Interaziendale A.O.U. Città della Salute e della Scienza di Torino, Turin, Italy.

14. Comitato etico della Provincia di Bergamo, Ospedal Papa Giovanni XXIII Bergamo, Italy

15. Comitato Etico Milano Area 3, ospedale Niguarda, Milano, Italy

16. Comitato Etico Provinciale di Brescia, Spedali Civili di Brescia, Brescia

17. Comitato Etico, Università degli Studi di Milano-Bicocca, Monza, Italy

18. Comitato Etico dell’A.O. San Giovanni di Dio e Ruggi d’Aragona, Salerno, Italy

19. Comitato Etico Catania 2, ARNAS Garibaldi Hospital, Catania, Italy

20. Comitato Etico Regionale Marche di AOU Ospedali Riuniti di Ancona, Ancona, Italy

21. Comitato Etico delle Province di Chieti e Pescara e dell’Università degli Studi “G. D’Annunzio” di

Chieti-Pescara, Chieti, Italy

22. Comitato Etico AOU SUN e AORN dei Colli, Napoli, Italy

23. Comitato Etico Unico della Provincia di Ferrara, Ospedale di Ferrara, Ferrara, Italy
